# NsrR from *Streptomyces coelicolor* Is a Nitric Oxide-sensing [4Fe-4S] Cluster Protein with a Specialized Regulatory Function*

**DOI:** 10.1074/jbc.M115.643072

**Published:** 2015-03-14

**Authors:** Jason C. Crack, John Munnoch, Erin L. Dodd, Felicity Knowles, Mahmoud M. Al Bassam, Saeed Kamali, Ashley A. Holland, Stephen P. Cramer, Chris J. Hamilton, Michael K. Johnson, Andrew J. Thomson, Matthew I. Hutchings, Nick E. Le Brun

**Affiliations:** From the ‡ Centre for Molecular and Structural Biochemistry, School of Chemistry,; the § School of Biological Sciences, and; the ** School of Pharmacy, University of East Anglia, Norwich Research Park, Norwich NR4 7TJ, United Kingdom,; the ¶ Department of Chemistry, University of California, Davis, California 95616, and; the ‖ Department of Chemistry and Center for Metalloenzyme Studies, University of Georgia, Athens, Georgia 30602

**Keywords:** DNA-binding Protein, Iron, Iron-Sulfur Protein, Nitric Oxide, Nitrosative Stress, Spectroscopy, Low Molecular Weight Thiol, Regulator

## Abstract

The Rrf2 family transcription factor NsrR controls expression of genes in a wide range of bacteria in response to nitric oxide (NO). The precise form of the NO-sensing module of NsrR is the subject of controversy because NsrR proteins containing either [2Fe-2S] or [4Fe-4S] clusters have been observed previously. Optical, Mössbauer, resonance Raman spectroscopies and native mass spectrometry demonstrate that *Streptomyces coelicolor* NsrR (ScNsrR), previously reported to contain a [2Fe-2S] cluster, can be isolated containing a [4Fe-4S] cluster. ChIP-seq experiments indicated that the ScNsrR regulon is small, consisting of only *hmpA1*, *hmpA2*, and *nsrR* itself. The *hmpA* genes encode NO-detoxifying flavohemoglobins, indicating that ScNsrR has a specialized regulatory function focused on NO detoxification and is not a global regulator like some NsrR orthologues. EMSAs and DNase I footprinting showed that the [4Fe-4S] form of ScNsrR binds specifically and tightly to an 11-bp inverted repeat sequence in the promoter regions of the identified target genes and that DNA binding is abolished following reaction with NO. Resonance Raman data were consistent with cluster coordination by three Cys residues and one oxygen-containing residue, and analysis of ScNsrR variants suggested that highly conserved Glu-85 may be the fourth ligand. Finally, we demonstrate that some low molecular weight thiols, but importantly not physiologically relevant thiols, such as cysteine and an analogue of mycothiol, bind weakly to the [4Fe-4S] cluster, and exposure of this bound form to O_2_ results in cluster conversion to the [2Fe-2S] form, which does not bind to DNA. These data help to account for the observation of [2Fe-2S] forms of NsrR.

## Teaser

**Background:** NsrR family proteins are [2Fe-2S] or [4Fe-4S] cluster-containing global regulators.

**Results:** *Streptomyces coelicolor* NsrR regulates only three genes, and it is the [4Fe-4S] form of the protein that binds tightly to NsrR-regulated promoters.

**Conclusion:** [4Fe-4S] NsrR has a specialized function associated only with nitric oxide stress response.

**Significance:** Members of the NsrR family are most likely all [4Fe-4S] proteins.

## Introduction

Nitric oxide (NO) is a reactive, lipophilic radical that can freely diffuse into cells. At low (nanomolar) concentrations, NO functions principally as a signaling molecule (*e.g.* via the reversible coordination of NO to the heme group in soluble guanylate cyclase to facilitate vasodilation in higher eukaryotes) and more widely through the process of thiol *S*-nitrosation, a regulatory process well characterized in eukaryotes (1) but also now recognized in bacteria (2). At higher concentrations (micromolar), NO is cytotoxic due to its reactivity with a wide range of targets resulting in nitrosation of amino acids (*e.g.* tryptophan) (3), nitrosative DNA damage (4), and nitrosylation of protein metallocofactors, particular those containing iron-sulfur (Fe-S) clusters (5). This property is exploited by mammalian macrophages in response to infection by pathogenic bacteria (6). Non-pathogenic bacteria also encounter significant concentrations of NO, through the activity of denitrifying species but also through the internal generation of NO resulting from the reduction of nitrite by nitrate reductases (7) and by the bacterial NO synthase enzymes encoded by some Gram-positive soil bacteria (8).

In order to survive, bacteria need to be able to counter the deleterious effects of NO. As a result, many bacteria have evolved a suite of specific iron-containing proteins to sense NO. Although the bacterial regulators SoxR and FNR are involved in coordinating the cell's response to NO (9–11), the primary functions of these regulators lie in sensing superoxide/redox stress and O_2_, respectively. However, two recently discovered regulatory proteins in *Escherichia coli* appear to be dedicated to sensing NO. NorR senses NO directly through a non-heme iron center and responds by switching on expression of the flavorubredoxin NorVW to detoxify NO (12). NsrR has also been shown to sense NO in *E. coli* and to switch on a regulon of at least 60 genes (13), including *hmp*, which encodes an NO-detoxifying flavohemoglobin (14) that converts NO to nitrate (or nitrous oxide under anaerobic conditions). This suggested that NsrR is a global regulator of NO-induced stress, whereas NorR has a more specific role in NO detoxification and a small regulon of only three genes, *norR-VW* (15).

NsrR belongs to the Rrf2 superfamily of regulators that includes the Fe-S cluster biosynthesis regulator IscR (16). Sequence alignment of NsrR proteins from a range of organisms revealed three conserved cysteine residues (Cys-93, Cys-99, and Cys-105 in *Streptomyces coelicolor* NsrR) in the C terminus region that probably act as cluster ligands (17). Consistent with this, Cys to Ala substitutions in *Neisseria gonorrhoeae* NsrR relieved repression of a target promoter and reduced DNA binding activity *in vitro* (18). Purified NsrR from *S. coelicolor* (19), *N. gonorrhoeae* (18), and *Bacillus subtilis* (20) have all been shown to be Fe-S cluster-binding proteins. However, the nature of the cluster and the mechanism by which the protein functions to coordinate the response to NO stress are not clear. Our studies are focused on NsrR from *S. coelicolor*, a model organism for the genus *Streptomyces*, which are widespread saprophytic soil bacteria that produce more than half of all known antibiotics and belong to the high GC Gram-positive phylum Actinobacteria. *S. coelicolor* is an obligate aerobe and encodes two homologues of flavohemoglobin, HmpA1 (SCO7428) and HmpA2 (SCO7094). The gene encoding one of these homologues (HmpA1) is adjacent to the gene encoding NsrR (SCO7427). Initial aerobic purification of *S. coelicolor* NsrR (produced in *E. coli*) resulted in a [2Fe-2S] cluster form (19) that was found to bind specifically to the *S. coelicolor hmpA1* and *hmpA2* promoter regions. This was consistent with data for *N. gonorrhoeae* NsrR, which suggested that it also contains a [2Fe-2S] cluster (18). However, anaerobically purified *B. subtilis* NsrR was found to contain a [4Fe-4S] cluster (20) and was recently shown to bind the *B. subtilis nasD* (nitrite reductase) promoter in an NO-sensitive manner (21). Therefore, the current literature on NsrR does not provide a consistent view of the nature of the Fe-S cluster.

Here we report ChIP-seq analysis to define the *S. coelicolor* NsrR regulon and DNase I footprinting and EMSA studies that confirm the target promoters and binding site. Spectroscopic and native mass spectrometry studies of anaerobically purified NsrR are also described, which, together with DNA binding studies, establish the physiologically relevant form of NsrR. These also reveal conditions under which facile cluster conversion occurs, accounting for the observation of different cluster types in purified NsrR proteins.

## EXPERIMENTAL PROCEDURES

### 

#### 

##### Strains, Plasmids, Cosmids, Primers, and Growth Conditions

The strains, plasmids, cosmids, and primers used in this study are listed in Table 1. *E. coli* was routinely grown on Luria-Bertani (LB) broth or agar or modified LB lacking NaCl to select for hygromycin resistance. *S. coelicolor* strains were grown on mannitol soya flour agar (20 g of mannitol, 20 g of soya flour, 20 g of agar in 1 liter of tap water), Difco nutrient agar (BD Biosciences). Liquid cultures were grown in Difco nutrient broth (BD Biosciences) or a 50:50 mix of Tryptone soy broth and yeast extract/malt extract (22).

**TABLE 1:** **Strains, plasmids, and primers used in this study**

Strains/plasmids	Description	Reference
**Strains**		
*S. coelicolor*		
M145	SCP1^−^ SCP2^−^ *S. coelicolor* wild-type strain	Ref. 22
JTM001	M145 Δ*nsrR*::*apr*	This work
JTM002	M145 Δ*nsrR* (unmarked)	This work
JTM003	JTM002 containing pJM001	This work
*E. coli*		
DH5α + BT340	Flp recombinase *E. coli* expression strain	Ref. 60
BW25113 (pIJ790)	*E. coli* BW25113 containing λ RED recombination plasmid pIJ790	Ref. 60
ET12567 (pUZ8002)	*E. coli* Δ*dam dcm* strain containing helper plasmid pUZ8002	Ref. 60

**Plasmid/cosmids**		
St5C11	*S. coelicolor* cosmid containing genes SCO7423–SCO7460	Ref. 61
St3A4.2.A04	*S. coelicolor* cosmid containing genes SCO7067–SCO7103 and a hygromycin-marked transposon-disrupting SCO7094	Ref. 26
pSET152	Integrative *Streptomyces* vector	Ref. 22
pGS21a	*E. coli* expression vector	Ref. 62
pNsrR	Expression construct for untagged native ScNsrR	Ref. 19
pJM001	pSET152 encoding NsrR with a C-terminal 3 × FLAG tag sequence	This work
pJM002	pGS21a encoding NsrR with a C-terminal His_6_ tag sequence	This work
pJM003	pJM002 containing a E85A mutation	This work
pJM004	pJM002 containing a D96A mutation	This work
pJM005	pJM002 containing a D113A mutation	This work
pJM006	pJM002 containing a E116A mutation	This work
pJM007	pJM002 containing a D123A mutation	This work
pJM008	pJM002 containing a D129A mutation	This work

##### ChIP-seq

Experiments were performed using a Δ*nsrR* mutant strain expressing a C-terminal 3×FLAG-tagged NsrR protein with the parent Δ*nsrR* strain as a control. The coding sequence for NsrR-3×FLAG was synthesized by Genscript with the native *nsrR* promoter and introduced into *S. coelicolor* Δ*nsrR* on the integrative vector pMS82 (23). 1 × 10^8^ spores of each strain were inoculated onto cellophane disks on mannitol soya flour agar plates (20 plates/strain) that were then grown for 48 h at 30 °C. The disks were removed and flipped so that the mycelium was submerged in 10 ml of a 1% (v/v) formaldehyde solution, within the Petri dish lids, for 20 min at room temperature to cross-link proteins to DNA. The disks were incubated in 10 ml of 0.5 m glycine for 5 min, and the mycelium was harvested, washed twice with 25 ml ice-cold PBS (pH 7.4), and incubated in 1 ml of lysis buffer (10 mm Tris-HCl, pH 8.0, 50 mm NaCl, 10 mg/ml lysozyme, 1× protease inhibitor (Roche Applied Science, complete mini EDTA-free tablets) at 25 °C for 25 min. Samples were then placed on ice, and 1 ml of IP^3^ buffer (100 mm Tris-HCl, pH 8.0, 250 mm NaCl, 0.5% (v/v) Triton X-100, 0.1% (w/v) SDS, 1× protease inhibitor) was added for 2 min prior to sonicating seven times at 50 Hz for 15 s each time. Material was centrifuged at 16,200 × *g* for 10 min at 4 °C, and supernatants were recentrifuged as above. A 1-ml sample was used for IP, 25 μl was used to prepare total DNA, and the excess was stored at −20 °C.

The 1-ml IP sample was precleared using 100 μl of equilibrated 50% (v/v) protein A-Sepharose beads and incubated at 4 °C for 1 h on a rotating wheel. Samples were then centrifuged at 16,200 × *g* for 15 min at 4 °C, and 100 μl of a 1 mg/ml solution of α-FLAG antibody (Sigma-Aldrich) was added, and the solution was incubated overnight at 4 °C on a rotating wheel. 100 μl of equilibrated protein A-Sepharose beads was added and incubated for 4 h at 4 °C on a rotating wheel. Samples were centrifuged at 1200 × *g* for 5 min and washed twice with 1 ml of 0.5× IP buffer for 15 min with gentle agitation and then twice with 1 ml of 1× IP buffer for 15 min with gentle agitation. Each sample was split into two 0.5-ml aliquots and centrifuged at 1200 × *g* for 5 min to remove all supernatant before 150 μl of elution buffer (50 mm Tris-HCl, pH 7.6, 10 mm EDTA, 1% (w/v) SDS) was added (or 10 μl for the total DNA samples) and incubated at 65 °C overnight. Tubes were inverted seven times and centrifuged at 16,200 × *g* for 5 min. Supernatants were retained, and the beads were washed with a further 50 μl of Tris (10 mm) and EDTA (1 mm) (pH 7.8) at 65 °C for 5 min before centrifuging at 16,200 × *g* for 5 min. Supernatants were pooled and centrifuged again at 16,200 × *g* for 1 min, and proteinase K (2 μl of a 10 mg/ml stock) was added to the supernatant and incubated at 55 °C for 90 min. Then 200 μl of phenol/chloroform was added, and samples were vortex-mixed for 3 min and then centrifuged for 3 min at 16,200 × *g*. The upper phase was stored, and the organic phase was re-extracted with 100 μl of Tris (10 mm) and EDTA (1 mm) (pH 7.8). Samples were then purified using a QIAquick kit (Qiagen), eluted with 50 μl of ultrapure water (Sigma), and re-eluted with the eluate. DNA was quantified using a nanodrop ND2000c spectrophotometer (Thermo Fisher), and libraries were constructed and sequenced by the Genome Analysis Centre (Norwich, UK).

##### Electrophoretic Mobility Shift Assays (EMSAs)

DNA fragments carrying the *hmpA1* (SCO7428), *hmpA2* (SCO7094), or *nsrR* (SCO7427) promoters were PCR-amplified using *S. coelicolor* genomic DNA with 5′ 6-carboxyfluorescein-modified primers (see Table 1). The PCR products were extracted and purified using a QIAquick gel extraction kit (Qiagen) according to the manufacturer's instructions. Probes were quantitated using a nanodrop ND2000c. The molecular weights of the double-stranded 6-carboxyfluorescein-labeled probes were calculated using OligoCalc (24). Band shift reactions (20 μl) were carried out in 10 mm Tris, 54 mm KCl, 0.3% (v/v) glycerol, 1.32 mm glutathione, pH 7.5. Briefly, 1 μl of DNA was titrated with aliquots of NsrR (20 μl final volume), typically to a 20-fold molar excess, and incubated on ice for ∼10 min. Loading dye (2 μl, containing 0.3% (w/v) bromphenol blue), was added and the reaction mixtures were immediately separated at 30 mA for 30 min on a 5% (w/v) polyacrylamide gel in 1× TBE (89 mm Tris, 89 mm boric acid, 2 mm EDTA), using a Mini Protean III system (Bio-Rad). Gels were visualized (excitation, 488 nm; emission, 530 nm) on a molecular imager FX Pro (Bio-Rad). Polyacrylamide gels were prerun at 30 mA for 2 min prior to use.

##### DNase I Footprinting

Footprinting was carried out as described previously (25) with the following modifications. DNA fragments carrying the *hmpA1* (SCO7428), *hmpA2* (SCO7094), or *nsrR* (SCO7427) promoters were PCR-amplified using the *S. coelicolor* cosmids 5C11 (*hmpA1* and *nsrR*) and 3A4 2.A04 (*hmpA2*) as templates (26, 27). In each case, one primer was end-labeled with ^32^P (PerkinElmer Life Sciences) using T4 polynucleotide kinase (New England Biolabs) in a 20-μl labeling reaction (2.5 μl of primer (10 pmol/μl), 11.5 μl of water, 2 μl of 10× T4 polynucleotide kinase buffer, 1 μl of T4 polynucleotide kinase, and 3 μl of γ-^32^P) incubated at 37 °C for 2 h and then 65 °C for 20 min. To this labeling reaction 30 μl of PCR mix was added (2.5 μl of second primer (10 pmol/μl), 1 μl of template (100 ng/μl), 1 μl of dNTP mix, 10 μl of 5× Q5 buffer, 10 μl of 5× GC enhancer, 5 μl of water, 0.5 μl of Q5 (supplied by New England Biolabs)), and thermal cycling conditions previously optimized using non-radiolabeled reagents were used. The subsequent PCR products were purified using QIAquick columns (Qiagen) according to the manufacturer's instructions. Binding reactions between DNA (∼100,000 cpm) and NsrR (0–2 μm) were carried out for 30 min at room temperature in 40 μl of reaction buffer (10 mm Tris, 54 mm KCl, 0.3% (v/v) glycerol, pH 7.5) before treatment with 10 units of DNase I (Promega) and 1 μl of 100 mm CaCl_2_ for 10–150 s. To terminate the reactions, 140 μl of stop solution (192 mm sodium acetate, 32 mm EDTA, 0.14% (w/v) SDS, 70 μg/ml yeast tRNA) was added and mixed by vortexing. Samples were extracted with 190 μl of phenol/chloroform, and the DNA-containing aqueous phase was ethanol-precipitated with 540 μl of 96% (v/v) ethanol. Pellets were dried and resuspended in 4 μl of loading dye (80% (v/v) formamide, 10 mm NaOH, 1 mm EDTA, 0.1% (w/v) xylene cyanol, 0.1% (w/v) bromphenol blue). A 6% (w/v) polyacrylamide sequencing gel with 8 m urea (Severn Biotech) was loaded with each sample in 1× TBE running buffer. The gel was maintained at 50 °C running at 1200 V to ensure uniform DNA denaturation and separation. Gels were transferred from glass plates to Whatman paper and dried for 30 min under vacuum. Labeled DNA was visualized using a PhosphorImager plate exposed for 16–24 h and scanned at 635 nm on a Typhoon FLA 9500 (GE Healthcare).

G+A ladders were produced based on the Sure track footprinting method. Labeled DNA (∼150,000 cpm) was incubated with 1 μg of poly(dI-dC) and 1 μl of 4% (v/v) formic acid for 25 min at 37 °C. Tubes were placed on ice, and 150 μl of fresh 1 m piperidine was added and incubated for 30 min at 90 °C. Reactions were cooled on ice for 5 min, and 1 ml of butanol was added to the mixture and vortexed vigorously. Samples were then centrifuged for 2 min, the supernatant was removed, and 150 μl of 1% (w/v) SDS and 1 ml of butanol were added and vortexed vigorously at room temperature. Reactions were then centrifuged for 2 min at room temperature, and pellets were washed two times with 0.5 ml of butanol (stored at −20 °C), centrifuging between washes at 4 °C. The supernatant was removed, and the pellet was checked using a Geiger-Müller counter. Pellets were dried for 5–10 min in a vacuum concentrator and then dissolved in 2–5 μl of loading dye (80% (v/v) formamide, 10 mm NaOH, 1 mm EDTA, 0.1% (w/v) xylene cyanol, and 0.1% (w/v) bromphenol blue).

##### Purification of S. coelicolor NsrR

Wild type NsrR was overproduced in aerobically grown *E. coli* strain BL21λDE3 cultures harboring pNsrR, as described previously (19). Cell pellets were washed with lysis buffer (50 mm Tris-HCl, 50 mm NaCl, 5% (v/v) glycerol, pH 7.1), transferred to the anaerobic cabinet, and stored at −10 °C in an anaerobic freezer (Belle Technology) until required. For Mössbauer studies, ^57^Fe (Goss Scientific)-labeled ScNsrR was produced *in vivo* as described previously (28). Unless otherwise stated, all subsequent purification steps were performed under anaerobic conditions inside an anaerobic cabinet (O_2_ < 4 ppm). Cell pellets were resuspended in lysis buffer with the addition of lysozyme (0.4 mg/ml), DNase I (1.3 μg/ml), 2 mm PMSF, and 1.3% (v/v) ethanol. The cell suspension was thoroughly homogenized by syringe, removed from the anaerobic cabinet, sonicated twice while on ice, and returned to the anaerobic cabinet. The cell suspension was transferred to O-ring sealed centrifuge tubes (Nalgene) and centrifuged outside of the cabinet at 40,000 × *g* for 45 min at 1 °C. The supernatant was passed through a HiTrap DEAE column (2 × 5 ml; GE Healthcare), and the eluate was immediately loaded onto a HiTrap heparin column (3 × 5 ml; GE Healthcare) and washed with lysis buffer until *A*_280 nm_ was ≤0.1. The heparin column was then washed with buffer A (50 mm Tris-HCl, 50 mm NaCl, 5% (v/v) glycerol, pH 8.0), and bound proteins were eluted (1 ml/min) using a linear gradient (20 ml) from 10 to 100% (v/v) buffer B (50 mm Tris, 2 m NaCl, 5% (v/v) glycerol, pH 8.0). Fractions (1 ml) containing NsrR were pooled, diluted 10-fold with lysis buffer, transferred to O-ring sealed centrifuge tubes (Nalgene), and centrifuged outside of the cabinet at 40,000 × *g* for 30 min at 1 °C. The supernatant was passed through a HiTrap DEAE column (5 ml) and immediately loaded onto a HiTrap heparin column (3 × 1 ml). The heparin column was then washed with buffer A containing 3% (v/v) buffer B and eluted using a linear gradient (2 ml) from 3 to 100% (v/v) buffer B. Fractions (1 ml) containing NsrR were pooled and stored in an anaerobic freezer until needed. Where necessary, gel filtration was carried out under anaerobic conditions using a Sephacryl S-100HR 16/50 column (GE Healthcare), equilibrated in buffer C (50 mm Tris, 100 mm NaCl, 5% (v/v) glycerol, pH 8) with a flow rate of 1 ml/min.

Protein concentrations were determined using the method of Smith (Pierce) (29) with bovine serum albumin as the standard. The iron and sulfide content of proteins were determined as described previously (30). This gave an extinction coefficient of ϵ_406 nm_ = 13.30 ± 0.19 mm^−1^ cm^−1^, which was subsequently used to determine the [4Fe-4S]^2+^ cluster concentration.

C-terminal His-tagged NsrR proteins (wild type and variants D85A, E96A, E113A, D116A, E123A, and E129A) were overproduced from pJM plasmids containing the SCO7427 sequence codon-optimized for *E. coli* (Genscript (Piscataway, NJ); see Table 1) in aerobically grown *E. coli* strain BL21λDE3, as described previously (28), except that 10 μm isopropyl 1-thio-β-d-galactopyranoside was used to induce protein expression. Cells were lysed in buffer C, as described above. The cleared cell lysate was loaded onto a HiTrap Ni^2+^ chelating column (2 × 5 ml), previously equilibrated with buffer C, and washed with 5% (v/v) buffer D (50 mm Tris, 100 mm NaCl, 200 mm l-histidine, 5% glycerol, pH 8.0). Bound proteins were eluted using a linear gradient (30 ml) from 5 to 50% (v/v) buffer D. Fractions (1 ml) containing NsrR were pooled, immediately loaded onto a HiTrap heparin column, and eluted with buffer B, as described above.

##### Preparation of [2Fe2S]-NsrR

An aliquot of [4Fe-4S] NsrR was diluted to a final concentration of ∼70 μm cluster with 20 mm Tris, 20 mm Mes, 20 mm Bistris propane, 100 mm NaCl, 5% (v/v) glycerol, 5 mm DTT, pH 8.7, containing dissolved atmospheric oxygen, and gently agitated for ∼50 min. The sample was immediately returned to the anaerobic chamber and buffer-exchanged (PD10 column, GE Healthcare) into phosphate buffer (50 mm potassium phosphate, 200 mm NaCl, pH 7.5). The sample was incubated at an ambient temperature for ∼5 min and then centrifuged at 14,100 × *g* for 2 min. The red pellet, containing [2Fe-2S] NsrR, was briefly washed with a minimal amount of phosphate buffer before being redissolved in buffer A containing 25 mm DTT. The supernatant, containing DTT-modified [4Fe-4S] NsrR, was discarded.

##### Preparation of Apo-NsrR

Native apo-NsrR was prepared from holoprotein using EDTA and potassium ferricyanide, as described previously (31), except that it was dialyzed against buffer A containing 5 mm DTT, and a HiTrap heparin column (5 × 1 ml) was used to isolate and concentrate the protein following dialysis. Briefly, the column was equilibrated with buffer A, and bound proteins were washed with 10 ml of buffer A containing 5.6 mm tris(2-carboxyethyl)phosphine and eluted using a linear gradient (20 ml) from 0% to 100% (v/v) buffer B.

##### Spectroscopy and Mass Spectrometry

UV-visible absorbance measurements were made with a Jasco V500 spectrometer, and CD spectra were measured with a Jasco J810 spectropolarimeter. Dissociation constants for the binding of low molecular weight thiols to [4Fe-4S] NsrR were determined by fitting plots of ΔCD_374 nm_ *versus* thiol concentration to a single site binding equation using Origin software (version 8; OriginLab, Northampton, MA).

2-(*N*-Acetylcysteinyl)amido-2-deoxy-d-glucopyranoside (dMSH) was prepared as described previously (32). A 13.87 mm dMSH stock solution was prepared and was determined to be ∼60% reduced (9.09 mm free thiol form) using a 5,5′-dithiobis(nitrobenzoic acid) assay (ϵ_412 nm_ ∼14,150 m^−1^ cm^−1^ (33)). To investigate the stability of the iron-sulfur cluster toward O_2_, aliquots of protein (∼10–45 μm cluster final concentration) and assay buffer (20 mm Tris, 20 mm MES, 20 mm Bistris propane, 100 mm NaCl, 5% (v/v) glycerol, pH 8.0) containing dissolved atmospheric O_2_ (234 ± 3 μm) were combined and mixed by inversion in a sealed cuvette outside of the anaerobic cabinet in the presence or absence of dithiothreitol. Loss of the iron-sulfur cluster was monitored at 406 nm as a function of time.

Resonance Raman spectra were recorded at 21 K using a scanning Ramanor U1000 spectrometer (Instruments SA, Edison, NJ) and an Innova 10-watt argon ion laser (Coherent, Santa Clara, CA), with 15-μl frozen droplets of sample mounted on the cold finger of a Displex model CSA-202E closed cycle refrigerator (Air Products, Allentown, PA). Laser power at the sample was 30 milliwatts, and the spectrum reported was the sum of 90 scans, with each scan involving photon counting for 1 s every 0.5 cm^−1^ and a spectral bandwidth of 7 cm^−1^. Mössbauer measurements were performed using an MS4 spectrometer operating in the constant acceleration mode in transmission geometry. The measurements were performed at 10 K using a Janis SVT-400 cryostat. 100 mCi of ^57^Co in rhodium held at room temperature was used as the source. Centroid shifts, δ, are given with respect to metallic α-iron at room temperature. The spectra were least square fitted using Recoil software (34).

For native MS analysis, His-tagged NsrR was exchanged into 250 mm ammonium acetate, pH 7.1, using Zeba spin desalting columns (Thermo Scientific), diluted to ∼6 μm cluster (6 pmol/μl), and infused directly (0.3 ml/h) into the ESI source of a Bruker micrOTOF-QIII mass spectrometer (Bruker Daltonics, Coventry, UK) operating in the positive ion mode. To study the effect of O_2_ and low molecular weight thiols, His-tagged NsrR was exchanged into ammonium acetate under anaerobic conditions. The resulting sample was diluted to ∼7 μm cluster with ammonium acetate buffer containing dissolved atmospheric oxygen (∼240 μm) and 5 mm DTT or 1.1 m β-mercaptoethanol. Full mass spectra (*m*/*z* 50–3500) were recorded for 5 min. Spectra were combined, processed using the ESI Compass version 1.3 Maximum Entropy deconvolution routine in Bruker Compass Data analysis version 4.1 (Bruker Daltonik, Bremen, Germany). The mass spectrometer was calibrated with ESI-L low concentration tuning mix in the positive ion mode (Agilent Technologies, San Diego, CA).

## RESULTS

### 

#### 

##### Identification of ScNsrR Binding Sites in Vivo

To determine where ScNsrR binds on the *S. coelicolor* chromosome, ChIP-seq analysis was carried out on 48-h, mannitol soya flour agar-grown cultures of the Δ*nsrR* strain with and without an NsrR-3×FLAG expression construct, integrated in single copy. ChIP was performed using monoclonal anti-FLAG antibodies, and immunoprecipitated DNA was sequenced using Illumina Hi-Seq.

**FIGURE 1.**
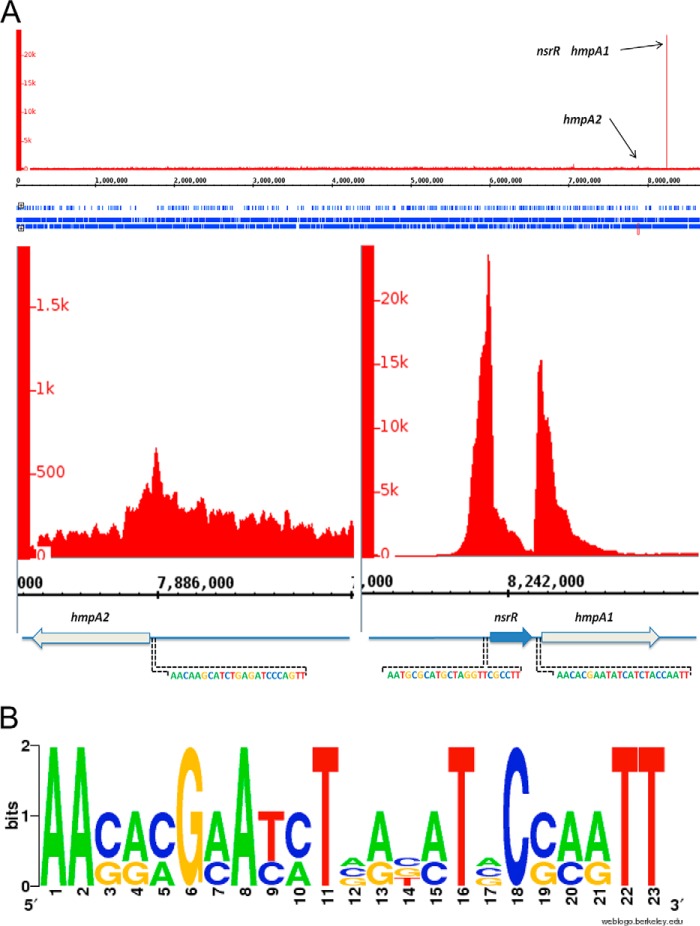
**Genes regulated by NsrR in *S. coelicolor*.** *A*, *top*, whole genome view of the ChIP-seq data for strain JM1002 (Δ*nsrR* expressing NsrR-3×FLAG), visualized using the Integrated Genome Browser (available on the BioViz Web site), showing only three enriched peaks when compared with the control strain JM1001 (Δ*nsrR*) that map to the *nsrR*, *hmpA1*, and *hmpA2* promoters. *Bottom*, the same JM1002 ChIP-seq data but *zoomed in* to view the *hmpA2, nsrR*, and *hmpA1* genes and the enrichment peaks at their respective promoters. The MEME-predicted ScNsrR binding site at each promoter is also shown. *B*, NsrR WebLogo generated by alignment of the three MEME-predicted NsrR sites at the *nsrR*, *hmpA1*, and *hmpA2* promoters.

The most significantly enriched DNA sequences in the NsrR-3×FLAG strain (compared with the control strain) mapped to the promoter regions of *hmpA1* and *nsrR* (Fig. 1*A*). This was surprising, because *hmpA1* is a weak match to the previously predicted NsrR binding site (35), and *nsrR* does not match at all. Furthermore, the *hmpA2* promoter, which shows a strong match to the predicted binding site, showed relatively low (<2-fold) enrichment in the ChIP-seq data (Fig. 1*A*), although it was previously shown to be bound by purified ScNsrR *in vitro* (19). Alignment of the *nsrR*, *hmpA1*, and *hmpA2* promoters using MEME identified a conserved sequence at all three promoters, and alignment of these sequences generated a 23-base pair consensus ScNsrR binding site, which consists of two 11-base pair inverted repeats separated by a single base pair (Fig. 1*B*). This binding site contains the DNA sequence previously shown to be bound by ScNsrR at the *hmpA1* and *hmpA2* promoters using AUC (19) but is significantly different in sequence to both the experimentally verified *E. coli* and *B. subtilis* NsrR binding sites (13, 20) and the predicted binding site for *Streptomyces* and Bacillales NsrR (35).

##### Anaerobic Purification of ScNsrR Results in a [4Fe-4S] Cluster-bound Dimer

**FIGURE 2.**
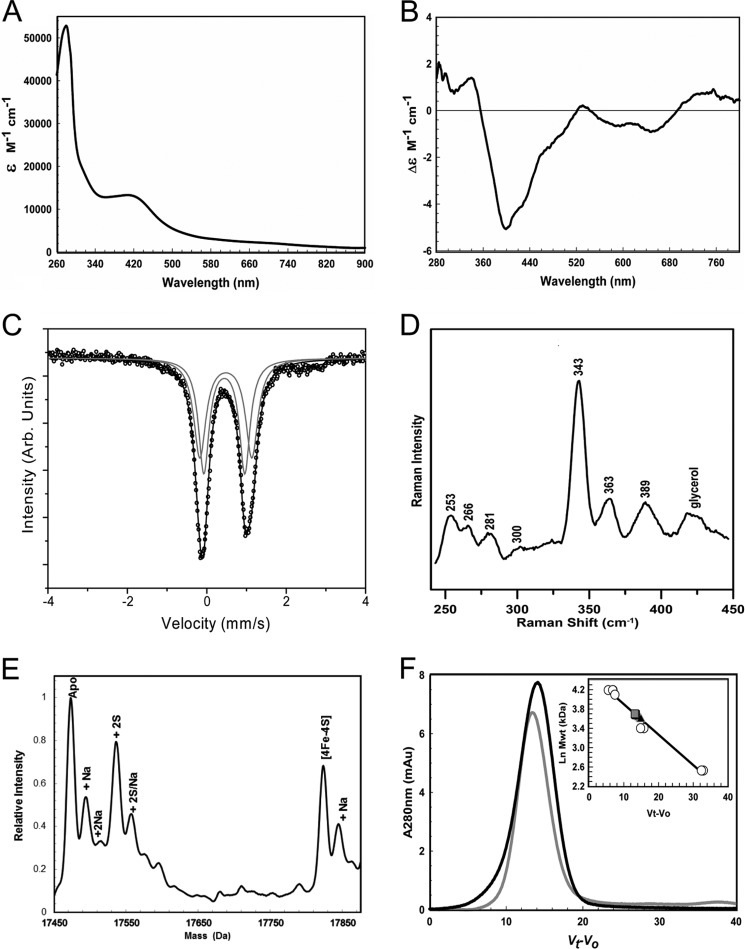
**Spectroscopic characterization of NsrR.** *A*, UV-visible absorption spectrum of 665 μm [4Fe-4S] NsrR, as isolated (∼60% cluster-loaded). *B*, CD spectrum of an identical sample. Extinction coefficients relate to the [4Fe-4S] cluster. The buffer was 50 mm Tris, 800 mm NaCl, 5% (v/v) glycerol, pH 8.0. *C*, Mössbauer spectrum of ∼0.75 mm [4Fe-4S] NsrR enriched with ^57^Fe. *D*, resonance Raman spectrum of ∼1.60 mm [4Fe-4S] NsrR. Excitation was at 488 nm, and temperature was 21 K. We note that the higher frequencies compared with those reported for *B. subtilis* NsrR are at least in part due to temperature difference (room temperature for *B. subtilis* NsrR) (20). The buffer was 50 mm Tris, 2 m NaCl, 5% (v/v) glycerol, pH 8.0, for *B* and *C*, respectively. *E*, positive ion mode ESI-TOF native mass spectrum of ∼7.5 μm [4Fe-4S] NsrR in 250 mm ammonium acetate pH 8.0. *m*/*z* spectra were deconvoluted with Bruker Compass Data analysis with the Maximum Entropy plugin. *F*, gel filtration analysis of NsrR association state. [4Fe-4S] (*black line*) and apo-form (*gray line*) samples of varying concentration (4–32 μm protein) were loaded in the presence or absence of DTT. *Inset*, calibration curve for the Sephacryl 100HR column. Standard proteins (*open circles*) were BSA (66 kDa), apo-FNR (30 kDa), and cytochrome *c* (13 kDa). [4Fe-4S]-NsrR and apo-NsrR are shown as a *black triangle* and *gray square*, respectively. The buffer was 50 mm Tris, 50 mm NaCl, 5% glycerol, ±2.5 mm DTT, pH 8.0. *mAu*, milliabsorbance units.

In order to validate the ChIP-seq data and analyze the ScNsrR binding sites at the three target promoters *in vitro*, it was necessary to purify the ScNsrR protein. Previous aerobic purification of ScNsrR in the presence of DTT, following overproduction in *E. coli*, resulted in a [2Fe-2S] form at a level of ∼30% cluster incorporation (19). A new strategy was devised to purify ScNsrR under anaerobic conditions and in the absence of any low molecular weight thiols (see “Experimental Procedures”). This resulted in a dark brown solution indicative of the presence of an Fe-S cluster. The UV-visible absorbance spectrum (Fig. 2*A*) revealed a broad absorbance band with a maximum at 406 nm (ϵ = 13302 ± 196 m^−1^ cm^−1^) and a pronounced shoulder feature at 320 nm. Broad weaker bands were observed in the 550–750 nm region. The spectrum is very similar in form to a number of [4Fe-4S] cluster-containing proteins (30, 31) and is quite distinct from that previously published for NsrR, which was characteristic of the redder color of a [2Fe-2S] cluster (19).

Because the electronic transitions of iron-sulfur clusters become optically active as a result of the fold of the protein in which they are bound, CD spectra reflect the cluster environment (36). The near UV-visible CD spectrum of NsrR (Fig. 2*B*) contained two positive features at 330 and 530 nm and a major negative feature at 400 nm, with smaller features at 570 and 640 nm. Although the sign of the band at 330 nm is reversed, the spectrum is otherwise similar to that of *S. coelicolor* WhiD, which contains a [4Fe-4S] cluster (31), and is again quite distinct from the previously published CD spectrum of NsrR (19).

Mössbauer spectroscopy provides definitive and quantitative determination of the type of iron-sulfur clusters present in a sample (37), so the spectrum of as-isolated, ^57^Fe-enriched NsrR was measured (Fig. 2*C*). The data fit best to two quadrupole doublets with similar isomer shifts (δ) and quadrupole splitting (Δ*E_Q_*), one having δ = 0.442 mm/s and Δ*E_Q_* = 1.031 mm/s and the other having δ = 0.481 mm/s and Δ*E_Q_* = 1.309 mm/s. Each doublet arises from a valence-delocalized [2Fe-2S]^+^ pair that couple together to form an *S* = 0 [4Fe-4S]^2+^ cluster (38). The isomer shifts and quadrupole splittings of both doublets are characteristic of [4Fe-4S]^2+^ clusters and are very similar to those reported for MiaB and lipoyl synthase, which both contain [4Fe-4S]^2+^ clusters that are coordinated by three Cys residues (39, 40). Furthermore, the Mössbauer parameters are markedly different from those of [2Fe-2S]^2+^ clusters, including that of IscR (41).

The low temperature (21 K) resonance Raman spectrum of NsrR (488-nm excitation) in the iron-sulfur stretching region (250–450 cm^−1^) is shown in Fig. 2*D*. The Fe-S stretching frequencies and relative resonance enhancements are characteristic of a [4Fe-4S]^2+^ cluster (42, 43) and are similar to those reported for *B. subtilis* [4Fe-4S] NsrR at room temperature (20). The bands are readily assigned by analogy with isotopically labeled model complexes and simple [4Fe-4S] ferredoxins under idealized *T_d_* or *D_2d_* symmetry (42), with mainly terminal Fe-S stretching modes at ∼389 and 363 cm^−1^ and mainly bridging Fe-S stretching modes at ∼389, 343, 300, 281, 266, and 253 cm^−1^ (both terminal and bridging Fe-S stretching modes are likely to contribute to the broad band at 389 cm^−1^). Previous studies of proteins have identified the frequency of the intense symmetric Fe-S stretching mode of the [4Fe-4S]^2+^ core as an indicator of ligation of a unique iron site by an oxygenic ligand, with all cysteinyl-ligated [4Fe-4S]^2+^ exhibiting frequencies spanning 333–339 cm^−1^ and those with one Asp or Ser ligand exhibiting frequencies spanning 340–343 cm^−1^ at low temperatures (≥77 K) (43, 44). Consequently, the high frequency of the symmetric bridging Fe-S stretching mode of the [4Fe-4S]^2+^ in NsrR (343 cm^−1^) is highly indicative of oxygenic ligation at a unique site of the [4Fe-4S]^2+^ cluster.

Native mass spectrometry was used to provide high resolution mass data of cluster-bound NsrR (see Fig. 2*E*). Here, a C-terminal His-tagged form of the protein was ionized in a volatile aqueous buffered solution that enabled it to remain folded with its bound cluster intact. The deconvoluted mass spectrum contained several peaks. The apoprotein was observed at 17,474 Da (predicted mass 17,474 Da), and there were adduct peaks at +23 and +64 Da due to Na^+^ (commonly observed in native mass spectra) and most likely two additional sulfurs (Cys residues of iron-sulfur cluster proteins appear to readily pick up additional sulfurs as persulfides (45)), respectively. The peak at 17,823 Da corresponds to the protein containing a [4Fe-4S] cluster with three deprotonated coordinating Cys residues (predicted mass = 17,474 − 3 + 352 = 17,823 Da). As for the apoprotein, peaks corresponding to Na^+^ and sulfur adducts of the [4Fe-4S] species were observed.

Previous studies of *S. coelicolor* NsrR revealed that the protein was a dimer in both [2Fe-2S] and apo-forms (19). The native mass spectrum of [4Fe-4S] NsrR did not reveal a dimeric form of NsrR. This may be because the dimeric form is not able to survive the ionization/vaporization process or because the protein is monomeric. To investigate this, anaerobic gel filtration of as-isolated NsrR (containing 60% holoprotein) was carried out. This gave a single elution band corresponding to a molecular mass of ∼37 kDa (see Fig. 2*F*). Removal of the cluster to generate a homogeneous apoprotein sample also gave rise to a single elution band at a mass of ∼40 kDa, consistent with the previous report (19).

The data presented here clearly indicate that under anaerobic conditions, the protein is isolated containing a [4Fe-4S]^2+^ cluster and is a homodimer, irrespective of the presence of a cluster.

##### [4Fe-4S] ScNsrR Binds Tightly to NsrR-regulated Promoters

**FIGURE 3.**
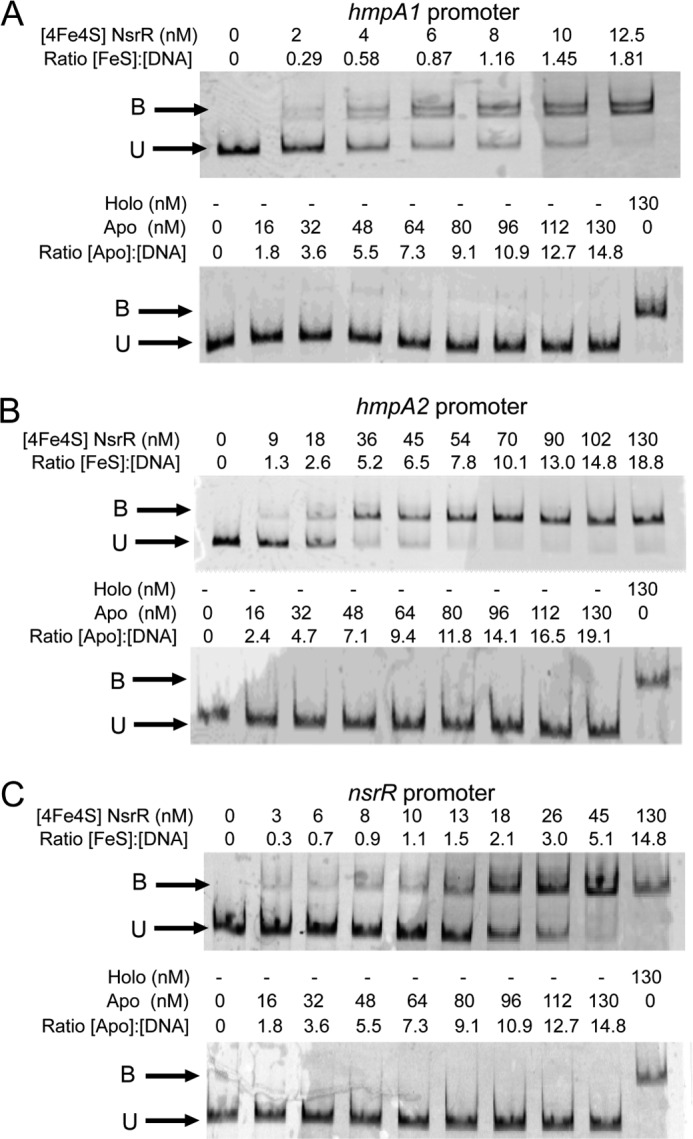
**Cluster-dependent DNA binding by [4Fe-4S] NsrR.** EMSAs using [4Fe-4S] or apo-NsrR (as indicated) and the *hmpA1* (*A*), *hmpA2* (*B*), and *nsrR* (*C*) promoters. Ratios of [4Fe-4S] NsrR to DNA are indicated. DNA concentration were 6.9 and 8.8 nm (*hmpA1* and *hmpA2*) or 8.8 and 8.8 nm (*nsrR*) for the [4Fe-4S] and apo-NsrR experiments, respectively. The binding buffer contained 10 mm Tris, 54 mm KCl, 0.3% (v/v) glycerol, 1.32 mm GSH, pH 7.5.

It was previously concluded that [2Fe-2S] ScNsrR binds to the *hmpA1* and *hmpA2* promoters (19) and the ChIP-seq data show that both *hmpA* promoters are bound by ScNsrR *in vivo* (Fig. 1*A*). Thus, it was of interest to investigate the binding properties of the [4Fe-4S] form with the same promoters and with the *nsrR* promoter, which we identified as an additional ScNsrR target using ChIP-seq (Fig. 1*A*). EMSA experiments were conducted with fluorescently (6-carboxyfluorescein) labeled PCR fragments carrying each of the three promoters, as described under “Experimental Procedures,” and the data for binding to *hmpA1* are shown in Fig. 3*A*. Increasing the concentration of as-isolated ScNsrR resulted in a clear shift in the mobility of the promoter DNA, and although the significance of the double band observed at low levels of ScNsrR is not known, the data demonstrate tight binding. The nature of the binding species is not completely clear because the ScNsrR sample contains both [4Fe-4S] and apo-forms, so a sample consisting entirely of apo-NsrR was also investigated. No evidence of binding was observed (Fig. 3*A*), demonstrating that the [4Fe-4S] cluster form of ScNsrR is the DNA-binding form of the protein. We conclude that the binding interaction between ScNsrR and the *hmpA1* promoter is tight, with full binding observed at a level of ∼2 [4Fe-4S] NsrR monomers per DNA. This is significantly tighter than previously reported for [2Fe-2S] NsrR, for which full binding of *hmpA1* DNA was not observed even with a several hundred-fold excess of protein (19). Data for the *hmpA2* and *nsrR* promoters are shown in Fig. 3, *B* and *C*, respectively. Binding of the [4Fe-4S] form was again observed, whereas the apo-form did not bind. For the *hmpA2* and *nsrR* promoters, full binding was observed at an excess of [4Fe-4S] NsrR over DNA of 8 and 5, respectively, indicating that ScNsrR binds the *hmpA1* promoter most tightly, consistent with the enrichment seen in the ChIP-seq experiment.

##### ScNsrR Binds to an 11-bp Inverted Repeat Sequence

**FIGURE 4.**
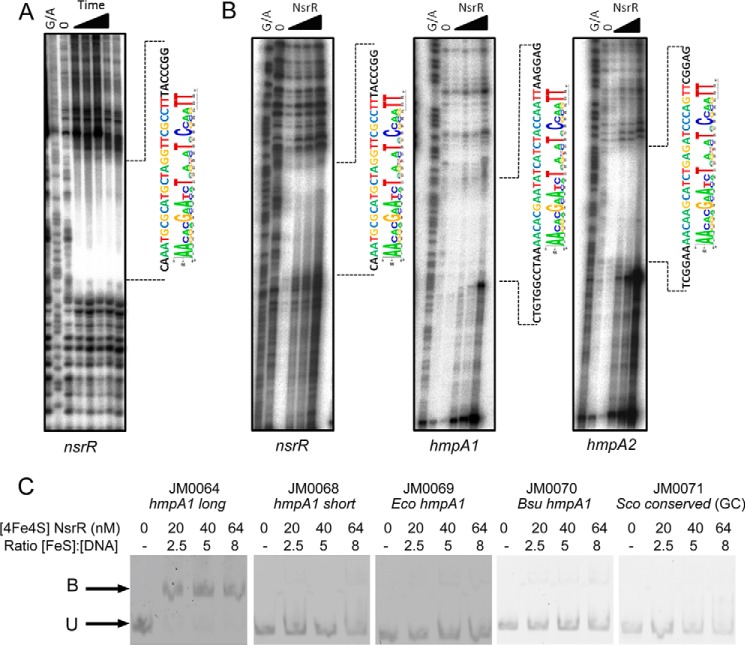
**DNase I footprinting and EMSA analysis of [4Fe-4S] NsrR binding to the *nsrR, hmpA1*, and *hmpA2* promoters.** *A*, footprint of NsrR bound to its own promoter. NsrR [4Fe-4S] at 2 μm was incubated with radiolabeled DNA for 0, 1, 2.5, 5, 7.5, and 10 min, respectively. *B*, footprints of increasing concentrations of NsrR bound to *nsrR*, *hmpA1*, and *hmpA2* promoters. The NsrR protein concentrations used were 0, 100, 250, 1000, and 2000 nm. *G/A*, Maxam and Gilbert sequence ladder. The regions protected by NsrR binding are indicated by *dotted lines*, and the sequence of the predicted binding site is shown *beside* the MEME-predicted consensus. *C*, EMSAs showing DNA probes bound (*B*) and unbound (*U*) by [4Fe-4S] NsrR. Probes used were JM0086, which has the conserved AA and TT removed from the ends of the binding site (*hmpA1 short*); JM0069, which contains the *E. coli hmpA1* binding sequence; JM0070, which contains the *B. subtilis hmpA1* binding sequence; and JM0071, in which the most conserved A/T base pairs have been changed to C/G. JM0064, containing the identified binding site (*hmpA1 long*) was included as a control.

MEME analysis revealed that all three ScNsrR target promoters contain a DNA sequence that resembles the 11-bp inverted repeat structure of the known NsrR binding sites in *E. coli* and *B. subtilis* (13, 46). To confirm that ScNsrR binds specifically to these sites at the *hmpA1*, *hmpA2*, and *nsrR* promoters, DNase I footprinting experiments were performed using ^32^P-labeled DNA fragments carrying each promoter. When the *nsrR* promoter fragment was incubated with ScNsrR and subjected to different DNase I digestion times the footprint was clearly visible (Fig. 4*A*), and this was confirmed in a separate experiment in which all three promoter fragments were incubated with increasing concentrations of ScNsrR before the addition of DNase I (Fig. 4*B*). The results clearly show a protected region covering the predicted binding site at each of the three promoters.

To probe important features of the binding site, additional EMSAs were performed. Deletion of the conserved AA and TT from either end of the *hmpA1* binding site, to make a truncated 19-bp site, abolished binding by ScNsrR, indicating that these conserved features are essential for recognition by ScNsrR (Fig. 4*C*). Similarly, substitution of all of the conserved A:T base pairs within the 23 bp site by C:G also abolished binding, suggesting that the unusual AT-rich features of the binding site are essential for recognition by ScNsrR (Fig. 4*C*). Probes carrying the experimentally verified NsrR binding sites from the *E. coli* and *B. subtilis hmpA* promoters were only very weakly bound by ScNsrR, indicating that the differences in DNA sequence are crucial for tight and specific binding of the NsrR proteins from these distantly related species (Fig. 4*C*).

##### [4Fe-4S] ScNsrR Binding to DNA Is Abolished by Reaction with NO

**FIGURE 5.**
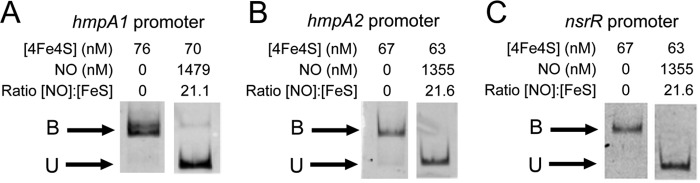
**Nitrosylation of NsrR [4Fe-4S] cluster abolishes DNA binding.** EMSAs using [4Fe-4S] before and after the addition of excess NO (as indicated) and the *hmpA1* (*A*), *hmpA2* (*B*), and *nsrR* (*C*) promoters. DNA concentrations were 10.6 nm (*hmpA1*), 5.9 nm (*hmpA2*), and 4.6 nm (*nsrR*). The binding buffer contained 10 mm Tris, 54 mm KCl, 0.3% (v/v) glycerol, 1.32 mm GSH, pH 7.5.

Exposure of [4Fe-4S] NsrR to a ∼20-fold excess of NO resulted in loss of binding to all three NsrR-regulated gene promoter regions (see Fig. 5). Thus, the high affinity DNA binding exhibited by [4Fe-4S] NsrR is sensitive to NO, consistent with its role as an NO sensor. Further details of the [4Fe-4S] cluster nitrosylation reaction will be described elsewhere.

##### Identification of the Non-Cys Ligand in [4Fe-4S] ScNsrR

**FIGURE 6.**
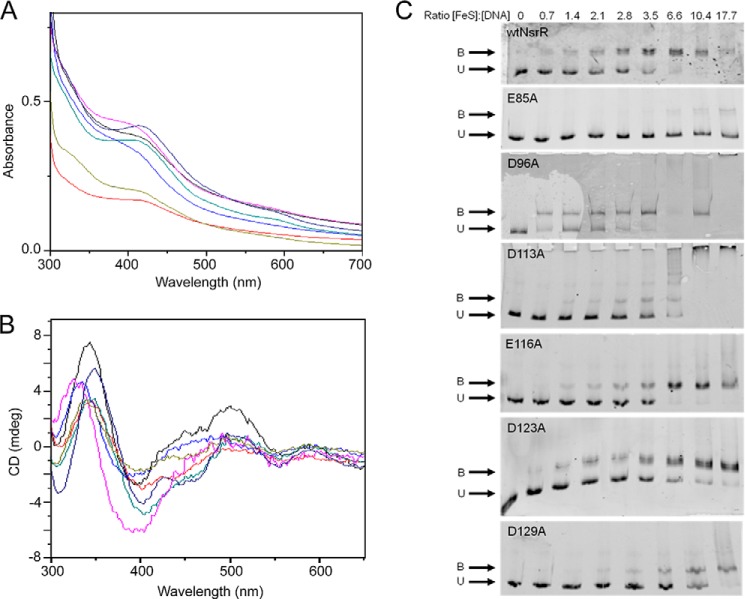
**Spectroscopic and DNA binding properties of NsrR site-directed variants.** Shown are overlaid UV-visible absorbance (*A*) and circular dichroism (*B*) spectra of NsrR variant proteins: E85A (*black*), D96A (*red*), D113A (*royal blue*), E116A (*cyan*), D123A (*magenta*), D129A (*olive green*). The spectrum of wild-type NsrR (*navy blue*) was included for comparison. Spectra were not corrected for concentration (path length, 1 cm). The absorbance spectrum of E85A was magnified ×5 to enable comparison; the CD spectra of D96A and D123A were magnified ×3 and ×2, respectively. The buffer was 50 mm Tris, 800 mm NaCl, 5% (v/v) glycerol, pH 8.0. *C*, EMSAs for site-directed variants of NsrR, as indicated, with wild-type NsrR shown for comparison. The *hmpA1* promoter was used as the DNA probe at concentrations of 15.1 nm (E85A and D123A), 14.5 nm (D96A), and 7.6 nm (wild type NsrR, D113A, E116A, and D129A). Ratios of [FeS] NsrR to DNA are as shown except for D96A wells 7 (empty) and 9 ([FeS]/[DNA] 20.8).

The resonance Raman spectrum of [4Fe-4S] ScNsrR indicated that an oxygenic ligand coordinates the cluster in addition to the three conserved Cys residues. Alignment of the characterized NsrR proteins from *E. coli*, *B. subtilis*, *Neisseria*, and *S. coelicolor* show that possible oxygenic ligands include Glu-85, Asp-123, and Asp-129, which are absolutely conserved, and Asp-96, Asp-113, and Glu-116, which are not conserved (17). A series of site-directed variants of NsrR was generated, in which carboxylate residues in these regions (which lie close to the three conserved Cys residues) were substituted by non-coordinating Ala. His-tagged variants E85A, D96A, D113A, E116A, D123A, and D129A ScNsrR were purified, and UV-visible spectra were recorded along with that of His-tagged wild-type ScNsrR (see Fig. 6*A*). Each protein was able to bind a cluster *in vivo* although at variable levels of incorporation and with somewhat variable absorbance properties. In particular, spectra due to E85A, D113A, and D123A NsrR were unusual in that absorption due to the cluster was shifted to higher energy (Fig. 6, *A* and *B*). The ability of the variant proteins to bind the *hmpA1* promoter region *in vitro* was investigated using EMSAs. The wild-type His-tagged protein, which fully bound DNA at a ratio of ∼6 NsrR/DNA, exhibited somewhat weaker binding than was observed for the non-tagged wild-type ScNsrR protein (Figs. 3*A* and 6*C*). The DNA-binding behavior of (His-tagged) D96A, E116A, D123A, and D129A NsrR proteins was similar to that of the tagged wild-type protein, with only minor variation in apparent affinities. Only the E85A and D113A ScNsrR proteins showed behavior different from that of wild-type ScNsrR. For D113A ScNsrR, specific DNA binding was observed, but as protein concentration increased, additional binding (as evidenced by a supershifted band) occurred, and, at the highest concentrations used here, aggregation occurred, with the protein and DNA remaining in the wells. Thus, although substitution of Asp-113 caused perturbations of the cluster environment, leading to aggregation at higher concentration, this variant was still able to bind specifically to DNA. This, alongside the fact that it is not conserved in other NsrR proteins, suggests that it is not a cluster ligand. In the case of E85A ScNsrR, however, there was no evidence of significant DNA binding, even at a ∼17-fold excess of protein, suggesting a significant loss of DNA binding activity (Fig. 6*C*). This loss of activity, combined with the fact that Glu-85 is well conserved, suggests that it may be the fourth ligand for the Fe-S cluster in NsrR.

##### Selective Interaction of Low Molecular Weight Thiols with [4Fe-4S] NsrR

**FIGURE 7.**
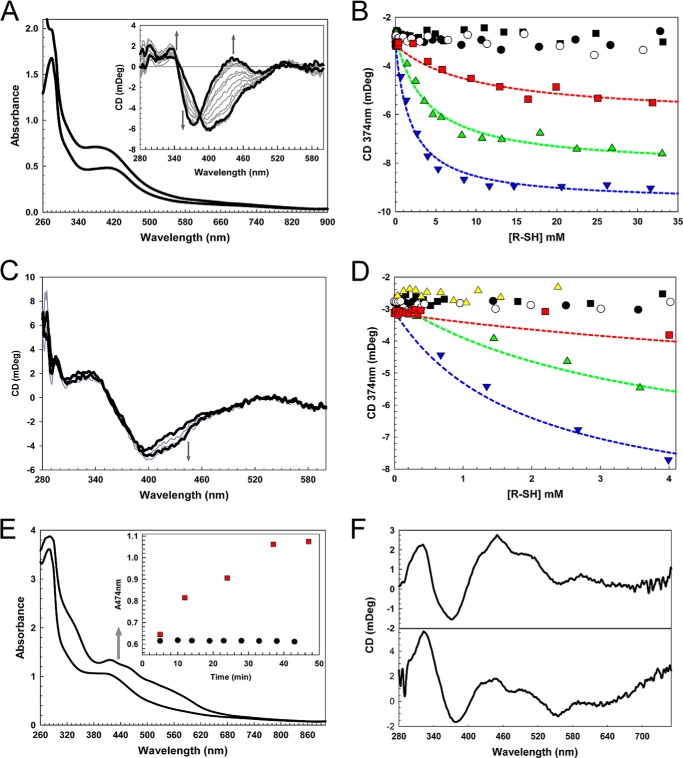
**Investigation of low molecular weight thiol binding to [4Fe-4S] NsrR and O_2_-mediated cluster conversion.** *A*, UV-visible absorbance spectrum of 36.3 μm [4Fe-4S] NsrR in the presence (*black line*) and absence (*gray line*) of 35 mm DTT. *Inset*, CD spectra resulting from a titration of an identical sample of [4Fe-4S] NsrR with DTT up to 35 mm. *Arrows* indicate the direction of spectra changes. *B*, changes in the CD spectrum (CD_374 nm_) in response to glutathione (*open circles*), l-cysteine (*black circles*), thiosulfate (*black squares*), DTT (*red squares*; *K_d_* = 9.9 mm), β-mercaptoethanol (*green triangles*; *K_d_* = 3.8 mm), and thioethane (*blue triangles*; *K_d_* = 1.9 mm). Fits to a simple binding equation (*dashed lines*) provide an estimate of the *K_d_* for each thiol. *C*, CD spectra of [4Fe-4S] NsrR titrated with *N*-acetylcysteine-glucose amine (dMSH). Minor changes between 400 and 460 nm suggest that dMSH may repair damaged FeS clusters in NsrR. *D*, changes in the CD spectrum (CD_374 nm_) in response to dMSH (*yellow triangles*) over a physiologically relevant concentration range (the responses due to other thiols shown in *B* are also plotted for comparison. The buffer was 20 mm Tris, 20 mm MES, 20 mm Bistris propane, 100 mm NaCl, 5% (v/v) glycerol, pH 8.0. *E*, absorption spectrum of NsrR exposed to O_2_ in the presence of 5 mm DTT for 5 and 47 min. The absorption spectrum is similar to that reported previously [2Fe-2S] NsrR (19). *Inset*, O_2_-induced absorbance changes at 474 nm in the presence (*red squares*) and absence (*black circles*) of 5 mm DTT. The buffer was 20 mm Tris, 20 mm MES, 20 mm Bistris propane, 100 mm NaCl, 5% (v/v) glycerol, pH 8.0. *F*, CD spectra of O_2_-modified NsrR (*top*) and [2Fe-2S] NsrR prepared as reported previously (19). The buffer was 50 mm Tris, 50 mm NaCl, 5 mm DTT, 5% (v/v) glycerol, pH 8.0.

It was recently reported that *B. subtilis* NsrR interacts with dithiothreitol (20), leading to the suggestion that low molecular weight thiols might be able to displace the non-Cys native ligand, resulting in all-thiolate coordination. To investigate whether ScNsrR also interacts with thiols, [4Fe-4S] ScNsrR was titrated with a range of low molecular weight thiols, including dithiothreitol, glutathione, and the more physiologically relevant mycothiol analogue *des-myo-*inositol mycothiol (dMSH), and visible CD spectra were recorded after each addition. Fig. 7*A* shows that dithiothreitol had a significant effect, with the major negative feature shifting to 374 nm with an isodichroic point at ∼382 nm. A plot of ΔCD_374 nm_ represents a binding isotherm, and fitting to a simple binding equation gave a *K_d_* of 9.9 mm (Fig. 7*B*), indicating a relatively weak interaction. Stronger binding was observed for β-mercaptoethanol (*K_d_* ∼3.8 mm) and thioethane (*K_d_* ∼1.9 mm) (see Fig. 7*B*). Glutathione, cysteine, and thiosulfate had no effect on the CD spectrum, indicating that they do not bind to [4Fe-4S] ScNsrR. Mycothiol (1-d-*myo*-inosityl-2-(*N*-acetylcysteinyl)amido-2-deoxy-α-d-glucopyranoside), an abundant low molecular weight thiol found at millimolar concentrations in most actinomycetes (47), serves as the major thiol redox buffer for *S. coelicolor*. dMSH is an analogue of mycothiol, only lacking the inositol group. The addition of dMSH caused only very minor changes in the spectrum (Fig. 7*C*), which were different in form from those above. A plot of ΔCD_374 nm_ over a physiologically relevant range (0–2.5 mm) (Fig. 7*D*) shows no evidence of dMSH binding. The observed changes suggest that dMSH may increase the [4Fe-4S] cluster content of ScNsrR, perhaps through promoting the repair of minor components of damaged cluster.

##### Thiol-mediated Conversion of the NsrR [4Fe-4S] Cluster to a [2Fe-2S] Form

As isolated, [4Fe-4S] ScNsrR is unreactive toward O_2_ (Fig. 7*E*, *inset*), with no loss of cluster observed up to 43 min after the addition of O_2_ and only 8% cluster loss observed after 120 min (not shown). Very different behavior was observed in the presence of 5 mm dithiothreitol, however. The addition of O_2_ resulted in a reddening of the color of the sample, and the UV-visible absorption and CD spectra of the resulting ScNsrR sample (Fig. 7, *E* and *F*) are very similar to those previously reported for [2Fe-2S] ScNsrR (19), consistent with the O_2_- and thiolate-mediated conversion of the [4Fe-4S] to a [2Fe-2S] form. The time course of the reaction (Δ*A*_474 nm_ *versus* time) (Fig. 7*E*, *inset*) shows that the conversion reaction was complete within 1 h. Similar experiments were conducted with β-mercaptoethanol and dMSH. In the presence of β-mercaptoethanol, [4Fe-4S] ScNsrR underwent a change similar to that observed with dithiothreitol, whereas dMSH had no effect on the O_2_ stability of the [4Fe-4S] cluster (not shown).

**FIGURE 8.**
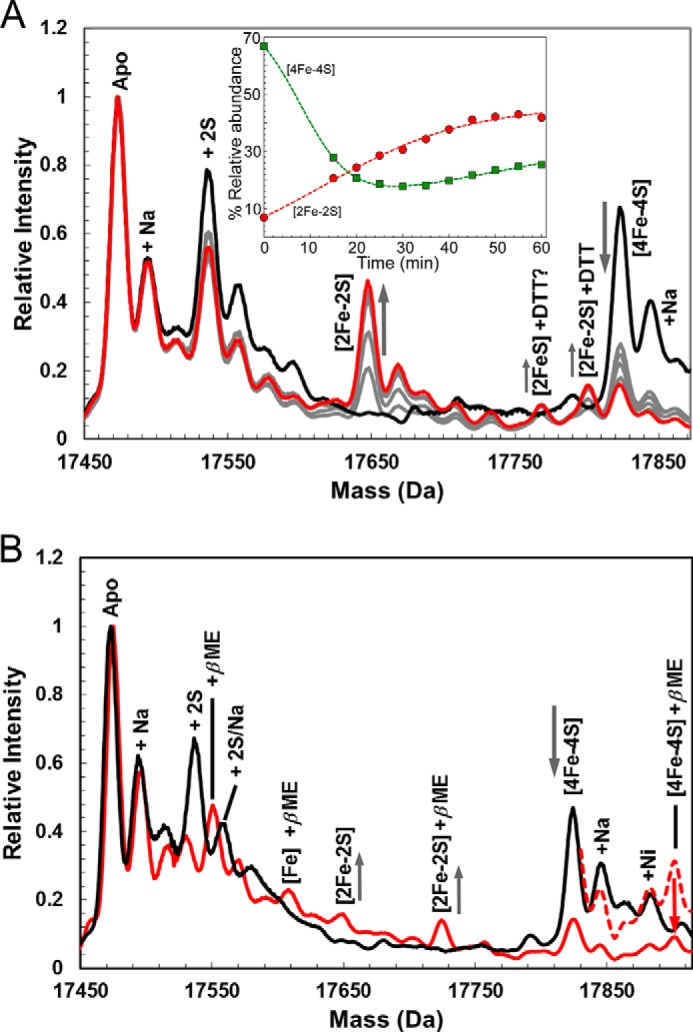
**Native MS analysis of O_2_- and low molecular weight thiol-induced cluster conversion of [4Fe-4S] NsrR.** Shown are ESI-TOF MS spectra of [4Fe-4S] NsrR (7 μm) in the presence of O_2_ (∼220 μm) and 5 mm DTT (*A*) and 1.1 m β-mercaptoethanol (*B*). Prior to the addition of thiol/O_2_ (*black line*), no [2Fe-2S] clusters were observed. In *A*, mass spectra were recorded at 0 min (*black line*); 15, 30, 45, and 55 min (*gray lines*); and 65 min (*red line*) postexposure. Plots of relative intensity of [4Fe-4S] and [2Fe-2S] NsrR as a function of time are shown in the *inset. Trend lines* are drawn in. In *B*, mass spectra were recorded at 0 min (*black line*) and 15 min (*red line*) postexposure. Prior to the addition of β-mercaptoethanol/O_2_ (*black line*), no [2Fe-2S] clusters were observed. After 15 min (*red line*, *dashed red line* multiplied ×3.5), β-mercaptoethanol adducts of [2Fe-2S] and [4Fe-4S] NsrR were observed. *m*/*z* spectra, recorded in the positive ion mode, were deconvoluted using Bruker Compass Data analysis software with the Maximum Entropy plugin. The buffer was 250 mm ammonium acetate, pH 8.0.

**FIGURE 9.**
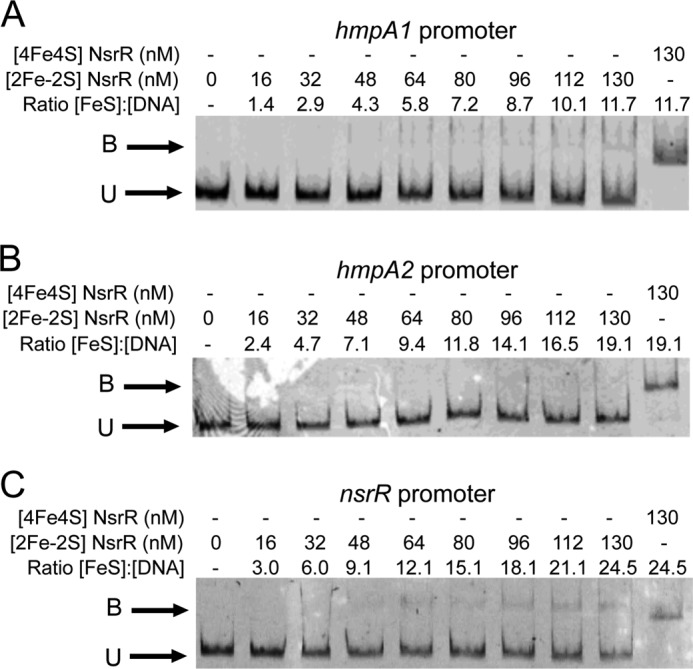
**DNA binding of [2Fe-2S] NsrR.** Shown are EMSAs using [2Fe-2S] NsrR (as indicated) and the *hmpA1* (11.1 nm) (*A*), *hmpA2* (6.8 nm) (*B*), and *nsrR* (5.3 nm) (*C*) promoters. Ratios of [FeS] NsrR to DNA are indicated. The binding buffer contained 10 mm Tris, 54 mm KCl, 0.3% (v/v) glycerol, 1.32 mm GSH, pH 7.5.

To investigate this further, native MS was employed. The addition of dithiothreitol to [4Fe-4S] ScNsrR resulted in the series of mass spectra shown in Fig. 8*A*. Over a period of 30 min, the peak at 17,823 Da due to [4Fe-4S] ScNsrR decreased, whereas a new peak at 17,647 Da was observed to increase in intensity. The new peak corresponds to ScNsrR with a [2Fe-2S] cluster bound to three deprotonated Cys residues (predicted mass = 17,474 − 3 + 176 = 17,647 Da). The small peak at 17,801 Da could be due to the [2Fe-2S] form bound by dithiothreitol (predicted 17,647 + 154 = 17,801 Da). Changes in relative intensity for the [4Fe-4S] and [2Fe-2S] forms are plotted as a function of time (Fig. 8*A*, *inset*). Similar experiments were conducted with β-mercaptoethanol, and similar effects were observed, with the [4Fe-4S] NsrR peak losing intensity and a [2Fe-2S] peak appearing (Fig. 8*B*). In this case, however, adducts of β-mercaptoethanol are more abundant, such that a β-mercaptoethanol-bound form of [2Fe-2S] ScNsrR, at 17,725 Da (predicted 17,647 + 78 = 17,725 Da) is more abundant than the [2Fe-2S] form. A β-mercaptoethanol-bound form of the [4Fe-4S] species at 17,901 Da (predicted 17,823 + 78 = 17,901 Da) was also observed. It is not absolutely clear that these are due to β-mercaptoethanol bound to the cluster, however, because a β-mercaptoethanol adduct is also detected for the apoprotein. However, together with the dithiothreitol experiment, the data are consistent with thiol binding of the cluster. To determine whether [2Fe-2S] ScNsrR binds to the promoter regions of *hmpA1*, *hmpA2*, or *nsrR*, EMSA experiments were repeated using a [2Fe-2S] ScNsrR sample treated to remove all traces of residual (non-converted) [4Fe-4S] NsrR (see “Experimental Procedures.” Fig. 9 shows that very little binding to *hmpA1* was observed, even at an excess of >10 [2Fe-2S] ScNsrR/DNA molecule. No evidence for binding to *hmpA2* was obtained even when [2Fe-2S] ScNsrR was present in 15-fold excess.

Overall, these data demonstrate that low molecular weight thiols that are able to bind to the cluster promote its reaction with O_2_, resulting in conversion to a [2Fe-2S] form in which the thiol may remain bound. This form of ScNsrR does not bind significantly to the *hmpA1* and *hmpA2* promoters.

## DISCUSSION

Anerobic purification of *S. coelicolor* NsrR resulted in a cluster-bound form of the protein that is different from that reported previously (19). Here we have demonstrated that this form of the protein contains a [4Fe-4S]^2+^ cluster and is a homodimer whether the cluster is present or not. The [4Fe-4S] form is stable to O_2_, consistent with the fact that *S. coelicolor* is an obligate aerobe, and binds tightly in a cluster-dependent manner to an 11-bp inverted repeat sequence in the promoter regions of *hmpA1*, *hmpA2*, and *nsrR*.

The relationship between [4Fe-4S] ScNsrR and the previously reported [2Fe-2S] form (19) was initially unclear. We noted that the resonance Raman spectrum of *B. subtilis* [4Fe-4S] NsrR was somewhat affected by the presence of dithiothreitol (with a decrease in the frequency of the symmetric bridging Fe-S stretching mode from 338 to 335 cm^−1^), and its reaction with O_2_ was markedly affected by the presence of dithiothreitol, resulting in a stabilization of the cluster and a mixture of [4Fe-4S] and [2Fe-2S] clusters (20). We have found that a number of low molecular weight thiols bind with low affinity to [4Fe-4S] NsrR, altering the spectroscopic properties of the cluster. All of the thiols that were found to bind (dithiothreitol, β-mercaptoethanol, and thioethane) are simple organic molecules with one or more thiol groups and no net charge. A number of thiols tested were found not to bind, and these were either more complex molecules with large substituents in addition to the thiol group (glutathione and dMSH) or were charged (thiosulfate). Thus, electrostatic and/or steric effects appear to be important for access of the thiol to the cluster site. Where binding was observed, the thiol was most likely able to compete for one of the iron sites, and it is also likely that this is the one that is not already coordinated by a thiol Cys. In those cases, the binding affinities reported here are better described as competition exchange constants rather than absolute binding constants, reflecting the competition between the natural ligand and the low molecular weight thiol.

Binding of an exogenous thiol to [4Fe-4S] NsrR was found to drastically reduce the O_2_ stability of the cluster, leading to rapid and stoichiometric conversion to a [2Fe-2S] form. These data explain why NsrR was previously characterized as a [2Fe-2S] cluster protein; in the original report, the protein was purified in the presence of dithiothreitol under aerobic conditions (19). Here, little or no binding of the [2Fe-2S] form to *hmpA1* and *hmpA2* promoters was detected. These observations appear to be inconsistent with the previous report of DNA binding by [2Fe-2S] NsrR. However, in those experiments, a several hundred-fold excess of [2Fe-2S] NsrR was present; in the current experiments, stoichiometric or near stoichiometric binding was observed for [4Fe-4S] NsrR binding to *hmpA1*, *hmpA2*, and *nsrR* promoters.

Thus, NsrR can accommodate either [4Fe-4S] or [2Fe-2S] clusters, and the O_2_-mediated conversion from the [4Fe-4S] to the [2Fe-2S] form is dependent on the presence of a coordinating low molecular weight thiol. Evidence from absorbance spectroscopy (where there was an increase in absorbance observed upon cluster conversion consistent with increased iron-thiolate coordination) and native MS (where [2Fe-2S] NsrR-thiol adducts were directly observed) suggests that the thiol remains bound to the [2Fe-2S] cluster and probably stabilizes it against further O_2_-mediated breakdown. Importantly, the physiologically relevant thiols l-cysteine and thiosulfate and the mycothiol analogue dMSH (48, 49) did not promote [4Fe-4S] to [2Fe-2S] cluster conversion. This suggests that cluster conversion is a result of *in vitro* protein handling, and we conclude that the [4Fe-4S] form of NsrR is the active form of the protein in the cytoplasm of aerobically growing *S. coelicolor* cells. However, given the facile nature of the cluster conversion reaction, albeit under specific conditions, we cannot rule out that this could have physiological significance in *Streptomyces* or other organisms. In the case of *S. coelicolor*, this would involve regulation of genes different from those identified here because we found no evidence of DNA binding for the [2Fe-2S] form.

Reaction of [4Fe-4S] NsrR with NO led to the loss of DNA binding, consistent with NsrR acting as an NO sensor. The data indicate that NsrR functions as a repressor under normal conditions. In the presence of NO, a conformational change must occur that disrupts DNA binding, resulting in derepression of genes encoding NO-detoxifying enzymes. The nature of the reaction with NO is currently under investigation, but it may be similar to the nitrosylation reactions of other [4Fe-4S] regulatory proteins, involving a rapid and complex reaction with up to eight NO molecules per cluster (10, 50).

NsrR proteins contain three conserved Cys residues that coordinate the cluster. Resonance Raman spectroscopy indicated that the fourth cluster ligand is oxygenic, and studies of site-directed variants highlighted two proteins with unusual properties (E85A and D113A), and of these only E85A showed no evidence of DNA binding. Furthermore, Glu-85 is totally conserved in experimentally verified NsrR proteins (17). Although we are not aware of an unambiguous example of cluster coordination by three Cys and one Glu residue, several instances of [4Fe-4S] clusters coordinated by three Cys residues and one Asp are known (*e.g. Pyrococcus furiosus* ferredoxin (43, 51), *Desulfovibrio africanus* ferredoxin III (52), and *B. subtilis* FNR (53). Therefore, Glu-85 is a reasonable candidate for the fourth ligand. Some caution is required, however, because substitutions of non-coordinating residues could *indirectly* affect the cluster environment and/or DNA binding properties of the protein. We note that the yield of variant E85A was much lower than for the other variants, and this could be a result of impaired stability. Therefore, although our data point to Glu-85 being the fourth cluster ligand, further confirmation is needed before a definitive assignment can be made, and this may require a high resolution structure.

Another well characterized member of the Rrf2 family of regulators, IscR, has been shown to bind a [2Fe-2S] cluster (41, 54). Although approximately 30% identity exists between IscR and NsrR, and the three Cys residues that coordinate the cluster are conserved, the spacing between them is not, and the fourth ligand to the cluster is different. For IscR, this was recently shown to be His-107 (41), a residue that is not conserved in NsrR proteins. The equivalent residue of Glu-85 in IscR is Asp-84, but substitution of this residue had no effect on IscR activity (41).

As clearly demonstrated here, NsrR appears to have inherent flexibility in its cluster-binding site, and IscR might share this flexibility. The variations in the nature and precise arrangements of coordinating ligands are likely to be important in determining the balance of stabilities between the different cluster forms.

Although IscR is arguably the best characterized Rrf2 protein, NsrR is the most widely conserved in the bacterial kingdom and has been characterized not just in Gram-negative gammaproteobacteria like *E. coli* K12, *E. coli* O157:H7, and *Salmonella* (13, 55, 56) but also in the Gram-negative betaproteobacteria *N. gonorrhoeae* and *Neisseria meningitidis* (18, 57), in the low GC Gram-positive Firmicute *B. subtilis*, and in the high GC Gram-positive actinomycete *S. coelicolor* (19). In all four branches of the bacteria, NsrR senses NO via an Fe-S cluster, and its primary function is to detoxify NO. *Neisseria* NsrR acts solely as a repressor and has a relatively small regulon of five genes, including *nsrR* (18, 57). It is somewhat unusual because it controls NO metabolism not via HmpA but by coordinating expression of the nitrite reductase (*aniA*) and NO reductase (*norB*) genes such that nitrite can be converted to nitrous oxide without a toxic build up of the intermediate NO. It has been argued that *Neisseria* strains are undergoing host adaptation by losing the ability to denitrify, through loss of *aniA*, and evolving into NO-tolerant aerobes (58). *Neisseria* NsrR also controls expression of *mobB*, which encodes an enzyme involved in molybdenum metabolism, and *dnrN*, which encodes a protein involved in repairing Fe-S cluster proteins damaged by nitrosative or oxidative stress (57). In *E. coli* and *B. subtilis*, NsrR regulates NO detoxification by controlling the production of HmpA. However, *E. coli* K12 NsrR regulates >60 target genes, and *B. subtilis* NsrR has a regulon of ∼35 target genes, many of which do not have an obvious role in NO metabolism (13, 46). *E. coli* and *B. subtilis* NsrR proteins regulate many of these target genes by binding to half-sites, but we could not detect any binding of ScNsrR to EMSA probes carrying artificial half-sites (not shown). This is consistent with the ChIP-seq analysis in which all three experimentally validated targets have full 11-bp inverted repeat binding sites. Intriguingly, in *E. coli* O157:H7, NsrR binds to full inverted repeat sequences at the promoters of the locus of enterocyte effacement LEE1 and LEE4 genes, and a half-site at the LEE5 promoter, all of which are on a chromosomal pathogenicity island. Bound NsrR activates these promoters by recruiting RNA polymerase, and activation is abolished by the addition of the NO releaser Nor-4 to cultures (56). To our knowledge, this is the only example of NsrR directly activating gene expression because all other reports describe it as a transcriptional repressor.

**FIGURE 10.**
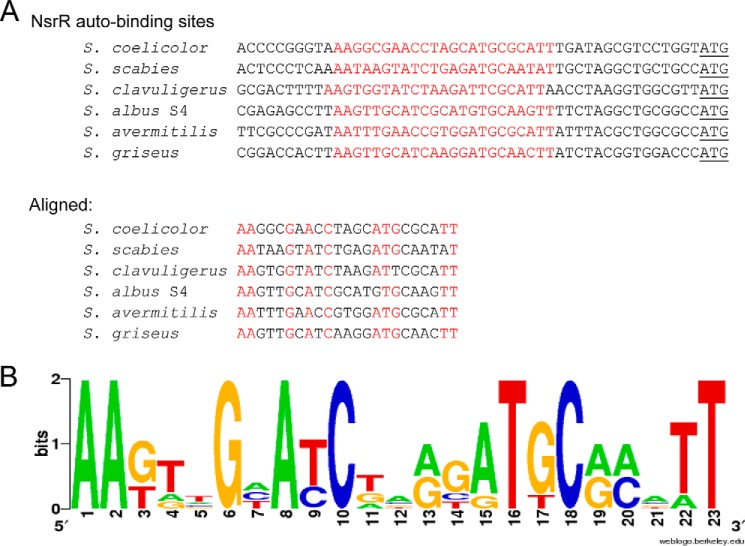
**Alignment of *nsrR* promoters.** *A*, alignment of *nsrR* promoters from six *Streptomyces* species (taken from StrepDB), revealing a conserved NsrR binding site at each. *B*, WebLogo generated by aligning these putative NsrR binding sites.

*S. coelicolor* NsrR has the smallest regulon reported to date and appears to be unique (thus far) in that its function is specialized to NO detoxification. Maintenance of an Fe-S-containing regulator to control its own expression plus that of two *hmpA* genes suggests that NO is a significant threat to *S. coelicolor* in its natural habitat. Of the complete genomes in the *Streptomyces* genome database, StrepDB, NsrR is conserved in all except for *Streptomyces venezuelae*, which also lacks an HmpA homologue but encodes a bacterial NO synthase enzyme. *S. venezuelae* may have lost NsrR-HmpA because it interferes with endogenous NO production via bacterial NO synthase. *Streptomyces scabies* also encodes bacterial NO synthase and has NsrR-HmpA, but the production of NO in *S. scabies* is tightly coupled to the biosynthesis of the phytotoxin thaxtomin (59). In the streptomycetes that encode NsrR, all of the *nsrR* genes contain a full NsrR binding site upstream of the translational start codon (Fig. 10), suggesting that autoregulation is a conserved feature.

## Footnotes

3 The abbreviations used are:

IP: immunoprecipitation
ScNsrR: *S. coelicolor* NsrR
Bistris propane: 1,3-bis[tris(hydroxymethyl)methylamino]propane
ESI: electrospray ionization
dMSH: 2-(*N*-acetylcysteinyl)amido-2-deoxy-d-glucopyranoside.
